# Newborn hearing screening in the limiar clinic in Porto Velho Rondônia

**DOI:** 10.1590/S1808-86942010000500012

**Published:** 2015-10-22

**Authors:** Marilia Silva e Nunes Botelho, Virgínia Braz da Silva, Luana da Silva Arruda, Isabel Cristiane Kuniyoshi, Lourdes Lebre Redes de Oliveira, Anderson Souza de Oliveira

**Affiliations:** 1 Consultant, Professor; 2 Consultant, Professor; 3 Speech and Hearing Therapist; 4 MSc; Professor; 5 Speech and Hearing Therapist; 6 Speech and Hearing Therapist. Faculdade São Lucas de Porto Velho - Rondônia

**Keywords:** hearing, infant, hearing loss, newborn.

## Abstract

**Abstract:**

With the universal hearing screening we can prevent auditory disorders in children.

**Aim:**

To characterize the program of neonatal auditory screening into a population of neonates.

**Materials and Methods:**

longitudinal cohort study. We surveyed the clinic's database on neonatal auditory screening in the city of Porto Velho, Rondônia.

**Results:**

Among the 6,889 newborns in the database, 5,700 (82.7%) passed and 1,189 (17.3%) failed the first screening. Of the group which failed 900 (75.7 %) returned for retesting. Among these, 15 (0.22 %) newborns had hearing loss confirmed. The most prevalent was neural hearing loss with 46.7% confirmed cases; they had hyperbilirubinemia as the most prevalent risk factor.

**Conclusion:**

hyperbilirubinemia was the most prevalent risk factor found in the group of hearing impaired children. The prevalence of hearing loss was of 2 in 1,000 newborns. It is important to highlight the relevant association between neural hearing loss caused by hyperbilirubinemia and sensorineural hearing loss of unknown causes.

## INTRODUCTION

Hearing may be the main means of contact men has with the environment, at least as far as basic interaction goes, which allows for the development of language and the intellect. For this reason, nature provided men with a noteworthy system to capture, amplify, perceive, discriminate and interpret sounds. It is in this order that the entire sound energy processing happens from the time it gets to the ear pinna, until it reaches the cerebral cortex^1^.

One of the main disorders which may impact language and speech development is hearing impairment. The American Speech-Language-Hearing Association considers that hearing impairment represents 60% of communication disorders^2^.

Hearing impairment can be classified as to the time when it occurs. Whether the hearing loss happens before or during birth - called congenital hearing loss; and when it happens after birth it is called acquired hearing loss^3^. Hearing loss is the most frequent and prevalent disorder found among those routinely screened in preventive health care programs^4^.

According to the Brazilian Committee of Hearing Loss in Children5, the incidence of significant bilateral hearing loss in healthy neonates is estimated to be between one and three neonates for every 1,000 births and in about 2 to 4 for every 1,000 babies coming from the Intensive Care Unit (ICU).

In 1994, the Joint Committee on Infant Hearing (JCIH)6 established the goal of universal detection of hearing loss in children through Universal Neonatal Hearing Screening (UNHS), and the development of studies to detect them, so as to make early intervention possible.

In 1998 the Universal Neonatal Hearing Screening Support Group (UNHSSG) was created, under the coordination of audiologist Mônica Jubran Chapchap, with the goal of making UNHS operational in Brazil. Since then, negotiations are under way in order to create a committee to draw UNHS resolutions^4^.

The first resolution of the Brazilian Committee on Hearing Loss in Children (BCHLC)^5^ was drawn on 11/29/1999 and one of its recommendations was to implement Universal Neonatal Hearing Screening (UNHS) for all children from birth to three months of age.

Routine Neonatal Hearing Screening (NHS) is the only strategy capable of early detection of hearing changes which can impact the individual's quality of life. Hearing changes detection process must start with neonatal hearing screening, being followed by diagnosis, hearing aid device fitting and early interventions. The first six months of life are decisive concerning the future development of children with hearing impairment^4^.

The American Academy of Pediatrics recommends the use of electrophysiological methods in NHS programs such as the Brainstem Auditory Evoked Potentials (BAEP) and Evoked Otoacoustic Emissions (EOAE) 6, 7, 8. In order to do an early diagnosis, newborns at risk for hearing impairment or not must undergo NHS in their first 48 hours of life, or before hospital discharge^4^,^5^,^7^.

The most employed and recommended technique for Neonatal Hearing Screening has been Transient Evoked Otoacoustic Emissions, since it uses low intensity acoustic stimuli, involving a broad range of frequencies and for being fast - taking about one minute per ear^9^.

BAEP aims at detecting mild to profound, conductive or sensorineural, uni or bilateral hearing losses. It is a non-invasive procedure, with proper specificity and sensitivity to examine newborns, reporting not only on the electrophysiological thresholds, but also the neurologic maturity as an important indicator, thus considering the growing number of preterm newborns found in the neonatal units^10^.

Diagnosis with early intervention can effectively contribute to improve the child's development when followed by a proper rehabilitation program^11^.

In order to achieve a quality monitoring of the neonatal hearing screening, it is necessary to have qualified professionals, control and calibration of the procedures, a database system, planning and evaluation of the procedures^12^.

Considering the lack of a neonatal auditory screening program in the city of Porto Velho, Limiar Clinic implemented the first NHS program in August of 2002, at the Dr. Ary Pinheiro Base Hospital. Limiar is a clinic certified and partner to the Public Health Care System (SUS), specialized in hearing diagnosis and rehabilitation, providing speech and hearing therapy for the state since August of 2001.

The NHS program team in Porto Velho was initially made up of two speech and hearing therapists and two secretaries, and there was no database implemented in order to manage the NHS data. Currently, the team counts on four secretaries, ten speech and hearing therapists, two ENT physicians, one social worker, one pediatrician, one neurologist and one psychologist. After many years we noticed the need to implement a database which would provide effective access to data and the follow up of those children who failed NHS.

The protocol used in the program has been suggested by the Universal Neonatal Hearing Screening Support Group (UNHSSG)^4^

The care for the newborn babies coming from the neonatal ICU and joint quarters happens before hospital discharge everyday of the week.

Given the aforementioned and the lack of epidemiological data on hearing impairment during childhood in the city of Porto Velho, the present study aimed at characterizing the neonatal hearing screening program developed at the Clínica Limiar in Porto Velho-RO.

## MATERIALS AND METHODS

This study was approved by the Ethics in Research Committee of the School under protocol # 322/08.

Study design: longitudinal historical cohort.

The data used in this study was collected from the Hearing Evaluation and Rehabilitation Clinic - LIMIAR, since this institution is responsible for neonatal hearing screening in the city of Porto Velho, state of Rondônia - Brazil. The data was collected from February of 2004 to October of 2006. Our sample involved 6,889 registered patients seen in the Dr. Ary Pinheiro Base Hospital.

We used the HITRACK 3.5 database software to manage NHS data. From this software we can obtain the number of newborns (NB) screened in joint quarters and those coming from the neonatal ICU, the number of NB who passed and failed the screening, number of newborns referred for diagnosis, risk indicators belonging to the registered newborns, number of NBs who were diagnosed with hearing impairment, their type and level of impairment.

The data collected was then submitted to statistical analysis, for which we used the Two-Proportions Equality Test, the Chi-Squared and the Fisher tests. We established 0.05 (5%) as significance level (how much error there may be in statistical conclusions, in other words, the statistical error of the analyses). All confidence intervals used throughout the study had 95% of statistical confidence.

## RESULTS

In our database we have 6,889 RNs registered, concerning the period from February of 2004 to October of 2006; 5,271 RN (76.5%) from joint quarters and 1,618 RN (23.5%) from the neonatal ICU. Of the 6,889 (100%) registered RNs, 5,700 (82.7%) passed the Transient Evoked Otoacoustic Emissions test and 1,189 (17.3%) were referred for retesting (Table 1).

**Table 1:** Distribution of the registered patients: pass/failure.

Registered	Joint Quarters		Neonatal ICU		Total	
	N	%	N	%	N	%
Passa	4294	62,3	1406	20,4	5700	82,7
Falha	977	14,2	212	3,1	1189	17,3
Total	5271	76,5	1618	23,5	6889	100

Legend N=absolute value; % = percentage;

ICU = Intensive Care Unit;

Among the 1,189 (100%) RNs who failed screening with OAEs, 900 (75.7%) came for retesting and 289 NBs (24.3%) did not (Table 2).

**Table 2:** Distribution of those who failed.

Registered	Joint quarters		Neonatal ICU		Total	
	N	%	N	%	N	%
Retested	702	59,5	198	16,65	900	75,7
Not retested	275	23,1	14	1,2	289	24,3

Legend N=absolute value; % = percentage;

ICU = Intensive Care Unit;

Of all 6,889 registered NBs (100%), 15 (0.22%) had their hearing loss confirmed, 2 (0.03%) coming from joint quarters and 13 (0.19%) from the neonatal ICU (Table 3). In our sample, we had a prevalence of 0.22% of hearing loss confirmed.

**Table 3:** Hearing Impairment Distribution.

Registered	Joint Quarters		Neonatal ICU		Total	
	N	%	N	%	N	%
Confirmed	2	0,03	13	0,19	15	0,22
Not confirmed	5269	76,47	1603	23,33	6874	99,8

Legend N=absolute value; % = percentage;

UTI = Intensive Care Unit;

Of the 15 NB (100%) who had hearing loss confirmed, 7 (46.7%) had neural hearing loss, 2 (13.3%) had conductive hearing loss and 6 NB (40%) had sensorineural hearing loss (Table 4). According to the statistical analysis, the percentage of neural hearing loss can not be considered different from that of the sensorineural hearing loss.

**Table 4:** Table 5 p-values

Confirmed HL	Central	Conductive
Conductive	0,046	
Sensorineural	0,713	0,099#

Equality Between Two Proportions Test

Legend HL = Hearing Loss; # p-values which because of being near the thresholds of acceptance are considered to have a tendency towards being significant.

Table 5 depicts the results regarding risk indicators for the 15 NB with confirmed HL, where we can see that the most prevalent risk indicator was hyperbilirubinemia, with five NBs (33.3%), followed by anoxia with two (13.3%), unknown cause with two NBs (13.3%), malformation with two NBs (13.3%), inheritance with one NB (67%), preterm/ low birth weight with one NB (6.7%), syndrome with one (6.7%) and one with congenital infection (6.7%). When we compare the hyperbilirubinemia risk indicator with the other indicators we notice that there was no statistically significant difference between them. Nonetheless, hyperbilirubinemia proved to be a statistically significant risk indicator (p-value<0.001) of neural hearing loss (Table 6).

**Table 5:** Risk Indicator Distribution.

Risk Indicator	N	%	p-value
Hyperbilirubinemia	5	33,3	
Anoxia	2	13,3	0,195
Unknown	2	13,3	0,195
Malformation	2	13,3	0,195
Inheritance	1	6,7	0,068#
Premature/low birth weight/hyperbilirubinemia	1	6,7	0,068#
Syndrome	1	6,7	0,068#
Congenital Infection	1	6,7	0,068#

Two Proportions Equality Test

Legend N=absolute value; % = percentage; # p-values for being near the acceptance thresholds tend to be significant.

**Table 6:** Association between Etiology and the Type of Loss

Etiology	Degree of Loss				General Total	
	Neural		Sensorineural			
	N	%	N	%	N	%
Anoxia	1	14,3%	1	16,7%	2	13,3%
Unknown	0	0,0%	2	33,3%	2	13,3%
Hyperbilirubinemia	5	71,4%*	0	0,0%	5	33,3%
General Total	6	60,0%	4	40,0%	10	100%

p-value = 0.034* Chi-Squared Test

Legend N=absolute value; % = percentage; ICU = Intensive Care Unit; *p-values = considered statistically significant considering the significance level used.

At the neonatal ICU we noticed a higher percentage of hearing impairment when compared to the NB coming from joint quarters, and such data was statistically significant (Table 7).

## DISCUSSION

The implementation of a HITRACK 3.5 database started in 2008 and, therefore, we are just starting to process the data from these six years of neonatal hearing screening.

According to the National Center for Hearing Assessment and Management Utah State University (NCHAM) an effective processing of the information is vital for the early detection and intervention on hearing impairment. Detection, diagnosis and intervention depend on the quick access to information, which is guaranteed by the HITRACK database^13^.

We noticed that the NHS program in Porto Velho/ RO followed the recommendations present in the literature, and the assessment and care for children with hearing loss are done by a multidisciplinary team^4^,^5^,^7^.

According to the Brazilian Committee on Hearing Loss in Children (BCHLC) 5, failure rates can vary between 5 and 20% when the screening is carried out with EOAEs on the first 24 hours of life, dropping to 3% when done between 24 and 48 hours of life. In the present study, 1,189 (17.3%) of the registered NB failed screening in the first stage of the NHS program (which happens before hospital discharge in the first 24 hours of life), matching literature data^5^,^7^.

A study carried out with 50 newborns during their first days of life and 80 during the second showed that on the second day of life there are significant improvements in the test and lower incidence of artifacts, concluding that the second day of life is a better time to perform the hearing screening in neonates with EOAE^14^.

In another study carried out in 2000 there was a significant improvement in the “pass” index of NHS after cleaning the external auditory meatus, and removing vernix. They also detected a reduction in tympanic membrane mobility in 22.7% of the ears tested, showing a significant effect on the “failure” index in hearing screening^15^.

In the present investigation, of the 1,189 NB (100%) who failed the first stage, 900 (75.7%) returned for retesting, showing a rate superior to those obtained in another study, which was of approximately 70%^16^. The program evasion index was of 289 NB (24.3%), this relevant data can be associated with the frequent instructions given by the professionals involved with children health to the parents/ guardians concerning NHS and the importance of hearing health for the development of language. Other authors^17^ noticed that the rate of evasion reduced from 85% to 25% after reinforcing the instructions given by all professionals involved with NHS.

The incidence of significant bilateral hearing loss in healthy neonates is estimated to be between 1 and 3 neonates for every 1,000 births^4^, ^5^, ^6^. Hearing loss is the most frequently found disorder in neonates, when compared to other disorders^7^, ^8^, ^9^. In our sample we had a prevalence of two neonates with hearing impairment confirmed for every 1,000 births, showing statistical rates similar to the ones found in the literature^4^, ^5^, ^6^ (Table 3).

In our study we noticed that of the 15 NB (100%) with confirmed hearing loss, the most prevalent type of HL was neural HL in seven NB (46.7%); however, according to the statistical analysis (Table 4), it cannot be considered different from the sensorineural hearing loss percentage found in six NB (40%). We've noticed that this percentage represents 0.1% of the neural hearing loss and 0.09% of the sensorineural hearing loss when compared to the total number of registered NB (6,889 NB). According to the literature, NBs with risk indicators for HL have greater likelihood of having sensorineural hearing loss^18^.

We found that the most prevalent indicator was hyperbilirubinemia, accounting for 33.3% of the cases, representing 0.09% of all registered NB. Newborns with high bilirubin levels who require blood transfusion have a higher risk of developing sensorineural hearing loss^10^.

A retrospective study involving 1,032 pediatric patients with hearing loss reported 67 cases (6.5%) of severe hyperbilirubinemia in the neonatal period, 30 of these patients had overt neonatal hyperbilirubinemia as the only risk indicator for hearing loss. In 26 of the 30 cases (87%) the otoacoustic emissions (OAE) were not present, while in the four remaining cases (13%) the emissions were detected, despite the absence of BAEP response^19^.

Often times, the cause of the auditory neuropathy is unknown, nonetheless, the following conditions may be associated with pediatric hearing neuropathy: hyperbilirubinemia, neurodegenerative disorders, hydrocephaly, and other diseases^20^. In the present study, hyperbilirubinemia proved to be a statistically significant risk in neural hearing loss (Table 6).

Many authors report that the children with the highest likelihood of having hearing loss upon screening are those with family history of congenital hearing loss, craniofacial malformation, low birth weight, anoxia, syndromes, ICU stay for more than 24 hours, hyperbilirubinemia and unknown etiology^4^,^5^,^8^,^10^. Such reports corroborate our findings, in which we observed the presence of hearing loss associated with unknown etiology (13.3%), anoxia (13.3%), craniofacial malformation (13.3%), inheritance (6.7%), preterm/low birth weight babies (6.7%) and congenital infection (6.7%).

In the joint quarters we identified two NBs with HL of unknown etiology, and such data was statistically significant, justifying the need for universal NHS^4^, ^5^, ^6^, ^7^, ^8^ (Table 6).

In the neonatal ICU we found a higher number of HL (13 NB) since it is a population with risk indicators for hearing loss. This information is statistically significant when compared to the results found from the joint quarters (Table 7).

**Table 7:** Hearing loss distribution.

Hearing loss	N	%	p-value
Joint quarters	2	0,03%	0,004*
ICU	13	0,19%	

Legend N=absolute value; % = percentage; ICU = Intensive Care Unit; *p-values= considered statistically significant considering the level of significance used.

## CONCLUSION

The rate of newborns who passed the first screening stage was of 82.7% (5,700) and 17.3% (1,189) failed it. We noticed that among the 1,189 NBs (100%) who were referred for retesting, 24.3% (289) did not come.

The hearing loss prevalence seen was of two NBs for every 1,000 births.

Hyperbilirubinemia has a higher prevalence among the risk indicators found among NB with confirmed hearing loss. We also noticed a statistically significant correlation between the neural hearing loss and the bilirubinemia risk indicator and sensorineural hearing loss with unknown etiology.

The risk indicators for hearing loss in cases diagnosed with hearing impairment were hyperbilirubinemia, unknown etiology, anoxia, craniofacial malformation, inheritance, prematurity/low birth weight, syndrome and congenital infection.

Based in the present study, we stress the need for using BAEP in the NHS of NBs with hearing loss risk and to follow these children with confirmed diagnosis of HL up. Health care professionals involved in NHS programs must use a database which may, in the future here in Brazil, help develop a multicentric study on pediatric hearing loss.
